# Efficacy of Selective Internal Radiation Therapy for Hepatocellular Carcinoma Post-Incomplete Response to Chemoembolization

**DOI:** 10.3390/ph16121676

**Published:** 2023-12-01

**Authors:** Salma Binzaqr, Frederic Debordeaux, Jean-Frédéric Blanc, Panteleimon Papadopoulos, Elif Hindie, Bruno Lapouyade, Jean-Baptiste Pinaquy

**Affiliations:** 1 Faculty of Medicine, University of Bordeaux, 33405 Talence, France; jean-frederic.blanc@chu-bordeaux.fr (J.-F.B.);; 2 Department of Nuclear Medicine, CHU Bordeaux, 33000 Bordeaux, France; frederic.debordeaux@chu-bordeaux.fr (F.D.); jbpinaquy@hotmail.com (J.-B.P.); 3 Department of Hepato-Gastroenterology and Oncology, CHU Bordeaux, 33000 Bordeaux, France; 4 Department of Diagnostic and Interventional Radiology, CHU Bordeaux, 33000 Bordeaux, France; panteleimon.papadopoulos@chu-bordeaux.fr (P.P.); bruno.lapuyade@chu-bordeaux.fr (B.L.)

**Keywords:** SIRT, HCC, TACE, SIR-Sphere, TheraSphere

## Abstract

Hepatocellular carcinoma (HCC) is one of the most common neoplasms worldwide and the third most common cause of cancer-related death. Several liver-targeted intra-arterial therapies are available for unresectable HCC, including selective internal radiation therapy (SIRT) and trans-arterial chemoembolization (TACE). Those two are the most used treatment modalities in localized non-operable HCC. TACE is the treatment option for patients with stage B, according to the BCLC staging system. In contrast, SIRT does not have an official role in the treatment algorithm, but recent studies showed promising outcomes in patients treated with SIRT. Although TACE is globally a safe procedure, it might provoke several vascular complications such as spasms, inflammatory constriction, and, in severe cases, occlusion, dissection, or collateralization. Hence, it is acclaimed that those complications could restrain the targeted response of the radio-embolization when we use it as second-line therapy post TACE. In this study, we will assess the efficacity of SIRT using Yttrium 90 Microspheres post incomplete or failure response to TACE. In our retrospective study, we had 23 patients who met the inclusion criteria. Furthermore, those patients have been followed radiologically and biologically. Then, we evaluated the therapeutic effect according to the mRECIST criteria, in addition to the personalized dose analysis. We found 8 patients were treated with TheraSphere^®^, with a median tumor absorbed dose of 445 Gy, while 15 received SIR-Spheres^®^ treatment with a mean tumor dose of 268 Gy. After radiological analysis, 56.5% of the patients had a complete response, and 17.3% showed partial response, whereas 13% had stable disease and 13% had progressive disease. For patients treated with SIRT after an incomplete response or failure to TACE, we found an objective response rate of 73.8%. Despite the known vascular complications of TACE, SIRT can give a favorable response.

## 1. Introduction

Hepatocellular carcinoma (HCC) is the sixth most common primary tumor worldwide and the fourth most common cause of cancer-related death. HCC occurs in the context of chronic liver disease (CLD), either due to viral hepatitis or alcohol abuse in more than 80% of cases [1]. Despite advances in prevention techniques, screening, and new modalities in diagnosis and treatment, the incidence and mortality of HCC continue to rise, with an estimate of the burden for this disease indicating more than 1 million annual deaths in 2030, according to the World Health Organization [2]. Chronic alcohol consumption is an important trigger for developing HCC, but it is not also limited to hepatitis B and hepatitis C, and non-alcoholic fatty liver disease is also a major risk factor for HCC.

Although an ongoing debate regarding the benefit of surveillance among high-risk individuals, a screening (by serum levels of alpha-fetoprotein (AFP) and ultrasound) every six months for HCC in those patients is recommended. The serum biomarker level of AFP does not confirm the diagnosis; however, it can be predictive of an eventual development of HCC, especially with a value superior to 400 ng/mL can confirm the diagnosis of HCC in 20% of patients. HCC is frequently diagnosed late in its course due to the absence of symptoms in patients in the early stage and the reluctance of some physicians to provide a screening for high-risk patients. The diagnosis of HCC can be made by clinical history, physical examination, and a standard noninvasive imaging modality such as ultrasound, MRI, and CT scan.

Radiological imaging techniques are of paramount importance in diagnosing HCC, which has characteristic features with an arterial hyperenhancement and venous washout in triphasic computed tomography or magnetic resonance imaging (which has a higher sensitivity than multidetector CT), according to the recent analysis of Lee et al. [3]. Several staging systems are available, but Barcelona Clinic Liver Cancer (BCLC) is endorsed as the optimal staging system and treatment algorithm, and it has been adopted widely due to its simplicity and prognostic reproducibility. An advanced HCC would complicate the underlying chronic liver disease or comorbidities that limit treatment options, particularly for tumors with segmental portal vein thrombosis, yet have a satisfying clinical situation with an accepted liver function. While evidence-based guidelines are immensely helpful, patient-specific characteristics and expertise centers are essential in implementing a personalized approach. Thus, finding the optimal treatment plan for those patients is challenging. In the setting of HCC, the tumor is supplied mainly from the hepatic artery, while the portal vein supplies the normal liver parenchyma. The keystone principle of regional treatment is targeted intra-arterial therapy. The locoregional therapy, particularly TACE, has been a mainstay in treating patients with unresectable HCC and an intermediate-stage BCLC for the past two decades. TACE is selective intra-arterial chemotherapy either with lipiodol (traditional) or drug-eluting microspheres (precision TACE, pTACE). However, its benefit is reduced in tumors larger than 7 cm [4]. Selective internal radiation therapy (SIRT) is an alternative intra-arterial therapy that primarily uses yttrium-90 (90Y) microspheres and relies on the delivery of radioactivity to destroy tumor tissue while sparing adjacent normal liver tissue. SIRT is not limited to HCC. It has also been broadly adopted as a locoregional therapy for intrahepatic cholangiocarcinoma and liver metastases of different malignancies, including neuroendocrine tumors (NETs) and colorectal cancer (mCRC).

Yttrium-90, which has been used in SIRT, is a pure beta-emitter with a half-life of 64.04 h without gamma-ray emission. However, it is possible to have a secondary bremsstrahlung photons emission that is useful for SPECT imaging, and it was used previously for post-therapy imaging, but it had a sub-optimal quality due to inefficient bremsstrahlung production, the continuous energy spectrum, and scatter penetration [5,6]. Positron emission is essential for TEP post-treatment imaging as it is by far superior to SPECT and considered a gold standard method. The maximum beta particle energy is 2.28 MeV, leading to maximum ranges in the soft tissue of 11 mm; 90Y could label resin microspheres (SIR-Spheres^®^), acrylic polymers bound to the carboxylic group on the surface, or embedded in glass microspheres (TheraSphere^®^). The therapeutic effect of SIRT is driven by a radiation effect, in contrast to the ischemia associated with chemoembolization.

SIRT has two phases of treatment; the first is angiography with intra-arterial injection of technetium-99m macro-aggregated albumin (99mTc-MAA) to evaluate the hepatic vasculature and exclude a major lung shunt. A 20% or higher lung shunt or any extrahepatic deposition can cause severe complications like ulceration or gastrointestinal bleeds that render the radioembolization contraindicated. Until now, SIRT does not have an established role in the treatment algorithm for HCC. However, several recent studies demonstrated a promising result, like Lewandowski et al., who reported a prolonged survival with 90Y over chemoembolization for patients with Child–Pugh A T3 and staged BCLC C [7]. Although TACE is a safe procedure globally, it can cause several vascular complications [8]. Hence, it is claimed that those complications could restrain the targeted response of SIRT when used as second-line therapy. As these treatments are potential therapeutic management for patients with HCC, it is crucial to determine whether one, in our case, TACE, does not prevent the use of the second or reduces its effectiveness within the therapeutic arsenal.

In keeping with recently published studies, our study evaluated the effectiveness of SIRT using yttrium-90 microspheres for unresectable HCC patients used as a second line of therapy after failure of partial response of a traditional TACE.

## 2. Results

### 2.1. Patients’ Characteristics

Twenty-three patients were included, with a male gender predominance: 19 patients were male and 4 were female, and the median age was 69 years (58–78 years). For risk factor analysis, 17 patients had liver cirrhosis due to alcohol abuse (*n* = 14), hepatitis B virus (*n* = 4), and NASH (*n* = 2). According to the BCLC staging system, 17 patients staged C (13 patients were due to confirmed portal vein thrombosis, three had suspected thrombosis so we considered them as positive, and two had hepatic hilar adenomegaly). In addition, four patients were in stage B, and two were in stage A. For the Child–Pugh scoring system, 21 patients were classified as A, whereas only 2 patients were classified as B with a score of 7. Eighteen patients had a single injection of SIRT, four patients had two injections, and two patients had three injections. Serological alpha-fetoprotein (AFP) was used as a biomarker in HCC, with a median of 84.4 (range 6–51,470).

Some patients had only one chemoembolization treatment, whereas others had several treatments ranging from one to four sessions. Then, the two SIRT phases are carried out, consequently, with a mean duration of 12.3 days (5–27 days). (Table 1 summarizes the patient characteristics.)

### 2.2. MAA-Based Dosimetry Analysis

The median pulmonary shunt fraction is 3.4% (range 0.96 to 8). Among the 23 patients, 8 were treated with TheraSphere^®^, and 15 were treated with SIR-Spheres^®^. The median injected activity was 1.104 Giga-Becquerel (GBq) for SIR-Spheres^®^ (IQR; 0.86–1.5 GBq) and 1.95 GBq (IQR; 1.41–2.545 GBq) for TheraSphere^®^.

### 2.3. 90Y-Based Dosimetry Analysis

**Figure 1:**
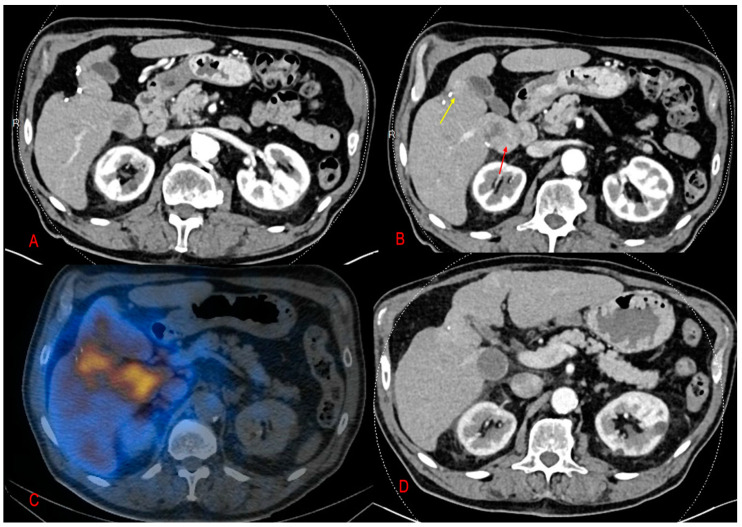
A 63-year-old man with recurrent HCC following tumor resection in segment V. (**A**,**B**) CT scan in arterial phase showing two hyper-vascular lesions, one in the segment I (red arrow) and the other beside the surgical clips (yellow arrow). (**C**) Fused images of PET-CT obtained 24 h following the treatment session of SIRT showed intense concentration of microsphere in the corresponding hepatic tumor (the orange content). (**D**) CT scan after five months of SIRT demonstrated a complete tumor regression.

**Figure 2:**
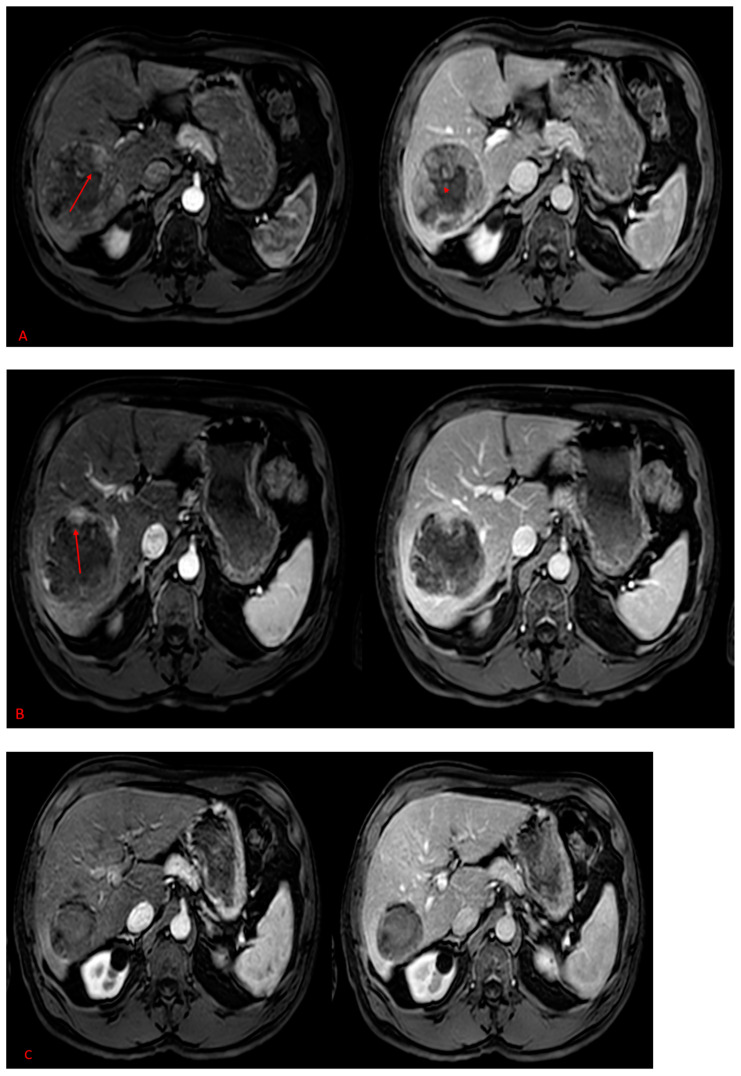
(**A**–**C**) A 73-year-old male with HCC in the right lobe, MRI images centered at the level of the celiac trunk, in different phases; arterial phase in the left and port venous phase in the right. (**A**) The initial MRI shows a large heterogeneous hypervascular tumor in the periphery with central necrosis. (**B**) After 2 TACE sessions, failure of the treatment, with persistence of enhancing viable remnant tissue as marked by the red arrow, compatible with residual disease. (**C**) MRI after six months of SIRT demonstrating a complete viable tumor regression.

The median tumor absorbed dose was 268 Gy (IQR; 107.1–243.2) for SIR-Sphere^®^ and 445 Gy (IQR; 349.5–563.5) for TheraSphere^®^. We then calculated the median absorbed tumor dose according to the tumor response. For the complete and partial response, we found a median absorbed dose of 167.4 Gy (IQR; 127.7–243.2) for SIR-Spheres^®^ and 520 Gy (IQR; 422.5–587.5) for TheraSphere^®^. In contrast, the median absorbed dose for the stable and progressive disease was 79.6 Gy (IQR; 50.7–183.7) for SIR-Spheres^®^ and 319 Gy (IQR; 288.5–349.5) for the TheraSphere^®^. Almost all patients tolerated SIRT well; apart from nausea and vomiting, no other significant side effect was reported. By analyzing those patients depending on our response criteria (mRECIST), 56.5% (*n* = 13) of patients showed complete response, and 17.3% (*n* = 4) had partial response. Figure 1 and Figure 2 are examples of patients who had a complete response. In contrast, 13% (*n* = 3) and 13% (*n* = 3) patients represented stable and progressive disease, respectively. (Table 2 summarizes the dosimetry analysis.) Three out of six patients categorized as having stable and progressive diseases were in 2015 when we did not yet start the personalized dosimetry protocol in our center. Thus, the injected dose was according to the standard approach, and it was eventually below the necessary dose.

## 3. Discussion

Our study is a retrospective monocentric study for patients with HCC. As HCC might be challenging in certain instances, the management needs a multidisciplinary approach. In France, for all cancerous diseases, the treatment plan should be discussed and approved during the RCP (réunions de concertation pluridisciplinaire), a medical reunion of physicians from different specialties, including oncologists, surgeons, radiotherapists, and nuclear medicine physicians to discuss a personalized treatment plan for each patient. According to the Organization of High Health Authority in France (HAS, Haute Auto-rité de Santé), SIRT was an alternative treatment option for patients in stage B of BCLC and selected cases in stage C with unresectable HCC and poor candidates for TACE, whether due to portal vein thrombosis, massive tumors, or bilobar disease [9]. Thus, after reclassification of BCLC in patients with failure or partial response of TACE, we have a high percentage of patients in stage C (75%). After discussing their situation in RCP, physicians decided to offer a locoregional treatment before starting systemic therapy. In particular, patients in stages B and C were able to benefit from complete financial coverage and reimbursement by the French national insurance.

While TACE is a globally safe procedure, certain studies have suggested that the hepatic artery post TACE may develop spasms, inflammatory constrictions, dissections, and thrombosis [8,9,10,11]. Maeda et al. [12] demonstrated the incidence, degree, and prediction of hepatic artery damage post TACE and concluded that about 16% of vascular damage is present per artery and about 48% per patient. Patients with no objective response post two TACE treatments were considered as a failure and would be less likely to benefit from another TACE but would benefit from an alternative therapy [13]. In the dilemma of those patients, selecting a treatment would be problematic whether to commence a systemic treatment or not. As reported in the TACTICS trial, that demonstrated a significantly higher PFS with TACE plus Sorafenib than TACE alone [14]. Nonetheless, SIRT is not yet supported by guidelines of HCC management; it could be an excellent alternative treatment due to its proven efficacy for patients who do not meet the curative treatment criteria and require tumor downstaging [15,16,17]. According to Moctezuma-Velazquez et al., who have evaluated SIRT in HCC across the different stages of BCLC, there is a median survival of 12.8 months for stage B and 9.3 for stage C, with only three months and half of difference that is worth the trial of SIRT in stage C [18]. Those studies were the key to attempting SIRT in certain patients with advanced HCC.

**Figure 3:**
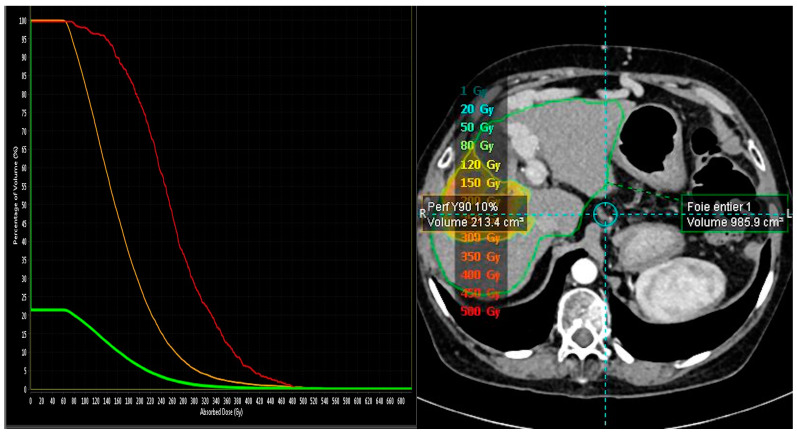
An example of dose volume histogram (DVH) computed with Simplicit90Y software. Abscissa is the absorbed dose; ordinate is the corresponding relative volume receiving the absorbed dose. The red curve represents the tumor, the orange curve represents the Y90 perfused volume, and the green curve represents the whole liver. As shown, 100% of tumor volume received a minimum of 60 Gy where the total absorbed dose was 485 Gy.

Concerning the dosimetry, in our study, we evaluated the tumor response in correlation with the personalized dosimetry to confirm the efficacity of SIRT. A personalized dosimetry is essential in evaluating the tumor-absorbed dose (TAD) and the non-tumor-absorbed dose (NTAD). Different retrospective studies revealed a complete tumor and radiographical response with doses of more than 205 Gy to the perfused volume. As reported by Salem et al., for TheraSphere^®^, a TAD ≥ 400 Gy is necessary for selective ablative to reach a pathologic necrosis, and only 300 Gy is necessary for downstaging or conversion to resection [19,20,21]. The impact of personalized dosimetry is not limited to response rate; it has a meaningful effect on overall survival, as demonstrated in the SARAH trial, which reported significant overall survival and disease control with a TAD ≥ 100 Gy for SIR-Sphere^®^ [10,22]. Nevertheless, we did not analyze the exact dosimetry of pre and post-treatment phases. Our software, Simplicit90Y™ (Version 2.4), approved in 2016 in Europe, has been used in several studies like the TARGET study published in 2021 [21]. A threshold absorbed dose is necessary to reach the radiation’s deterministic effects and have the desired response. The higher the dose above this threshold, the more severe the effect will occur. Therefore, the tumor absorbed dose is higher than the dose to global perfused volume due to a preferential blood flow to the tumor. On the other hand, one of the critical limitations of SIRT is the tolerance of normal liver parenchyma to radiation. Hence, it is essential to acknowledge the safety threshold dose with a limit of globally (30/40) 70 Gy for non-tumoral absorbed dose with SIR-Spheres and 75 Gy for TheraSphere [23]. As reported by the DOSIPHERE-01 trial, which compared standard dosimetry with personalized dosimetry for patients with hepatocellular carcinoma, they found that, according to EASL criteria, the objective response in personalized dosimetry was significantly higher in the personalized dosimetry group (71% versus only 36% for the standardized dosimetry group) [21]. So, our study followed the recommendation for personalized dosimetry, and we had a reasonable outcome. Through the post-treatment analysis, we can analyze the exact absorbed dose to the tumor, the perfused volume, and the whole liver. Figure 3 is a histogram of the post-therapeutic anaylsis of a patient with a complete response tumor, where we can see three different color curves; each curve represents a specific area with its absorbed activity, such as a tumor, perfused volume, and the whole liver.

In our extensive search in PubMed and Medline to identify peer-reviewed 241 clinical studies using the following terms—TheraSphere^®^, transarterial radioembolization, SIRT, and SIR-Spheres^®^—we found a similar study that has been published recently and they evaluated 90Y-SIRT for HCC refractory to prior trans-arterial embolization, or TACE, and they concluded that TARE has a response rate of 85% of patients, which is relatively close to our result [24].

Some antineoplastic agents are known to be potent radiosensitizers and could increase the effect of 90Y-microspheres. Nevertheless, this attitude must select patients wisely because this combination therapy may also increase adverse effects. Indeed, it is crucial to evaluate and quantify the benefit of SIRT when combined with chemotherapy and targeted systemic therapy. In this context, a study published in 2019 by Jens Ricke et al. concluded that there was no significant improvement in OS in patients with advanced HCC treated with SIRT, in addition to sorafenib compared with sorafenib alone [25]. No other recent studies evaluated the combination of SIRT with systemic treatment defined in the BCLC staging and treatment strategy, like Atzolizummab or Regorafenib.

Our study has limitations, including the small sample size and the retrospective nature. In addition, our collected data through a long period from 2015 to 2022 with a variant protocol application and treatment target in terms of complete resolution or downstaging for conversion treatment, which is a crucial point as it impacts the personalized dosimetry as well as the overall response. Despite these limitations, our study showed an interesting result that might be a potential point for a prospective study with a prohibitively large number of patients, a control group, and clear criteria for treatment protocol to have less biased results about SIRT used as a second line of treatment either alone or in comparison to other treatment like the systemic one.

## 4. Methods and Materials

### 4.1. Study Design

A retrospective, monocentric investigation was performed at Bordeaux University Hospital. We investigated 280 patient files from our database for the period running between January 2015 and May 2023, using the hospital software systems DxCare (Dedalus, Le Plessis-Robinson, France) and Xplore (EDL, La Seyne-sur-Mer, France). We obtained informed consent from each patient at the time of treatment. All procedures were performed in accordance with the ethical standards of our institutional and national research committee and the Declaration of Helsinki. We found 30 eligible patients who met the inclusion criteria: (1) a localized HCC not amenable to surgical resection; (2) treated previously by a traditional TACE, regardless of the number of sessions, with failure or partial response; and (3) subsequently retreated by SIRT following TACE within a minimum of a six-month interval of the same lesion(s).

Out of those 30 patients, we excluded seven patients. Two patients had two different hepatic malignancies (one had HCC and cholangiocarcinoma, and the other had colorectal metastasis and HCC). One did not undergo the second session of SIRT because of the deterioration of his clinical situation, and another had an interrupted injection during the second due to sudden severe pain. Also, one patient had radioembolization for a different lesion. One patient had a liver transplant two months post-SIRT, and finally, one patient lacked follow up.

### 4.2. Treatment Protocol

Then, we followed the radioembolization protocol, which consists of several steps: a pre-therapeutic angiography with intra-arterial injection of 99mTc-MAA, which is an essential and mandatory step to determine if the patient is eligible for SIRT by assessing the hepatic vasculature anatomy and exclude any extrahepatic flux or gastrointestinal shunting. Indeed, 99mTc-MAA is an acceptable surrogate for the future distribution of 90Y-microspheres. During the angiography, the radiologist decides how many needed injections according to the arterial territory of the tumor. Then, we did a 99mTc-MAA scintigraphy to evaluate the perfusion volume, quantify potential hepatopulmonary shunt, and calculate an individualized dose [23].

The dosimetric analysis defines the therapeutic approach, which may be segmental, sectorial, or lobal (whether unilobed or bilobal) depending on the tumor’s location and size. Simplicit90Y™ software (Mirada Medical LTD., Oxford, UK) was used for calculating the personalized dosimetry by integrating acquisitions of the perfused volume of 99mTc-MAA scintigraphy with either a CT or MRI images as well as the acquisition of the pre-therapeutic angiography. Moreover, we precisely contoured the tumor, the perfused volume, and the whole liver to calculate the required administered activity to reach a given absorbed dose except for three cases that were treated several years ago (in 2015) outside of the current personalized dosimetry recommendation where the personalized dosimetry system was yet unavailable in our center. Thus, the 90-Y activity for those three patients was administered according to the standard method by applying the partition model based on the MAA distribution and a threshold of 30 Gy for the non-tumoral liver. Then, after a few days of the first phase, patients underwent SIRT using 90Y-microspheres that were injected in the same catheter position selected during the 99mTc-MAA perfusion. Older patients were treated with resin microspheres, where other microspheres were unavailable. Later, patients were randomly treated with SIR-Sphere or TheraSphere regardless of the tumor characteristics. However, there was a preference for SIR-Sphere for bulky tumors as its size ranges between 20–60 μm compared to the small molecule size of Therasphere, which ranges between 20–30 μm. For 90Y microspheres, we included the two commercially available microspheres, SIR-Sphere^®^ (Sirtex Medical Limited, Sydney, Australia) [26] and TheraSphere^®^ (Boston Scientific, Marlborough, MA, USA) [27]. Patients underwent a post-therapeutic PET scan after the therapeutic phase, either on the same day or the next day, to verify the microspheres’ diffusion to the requested territory. Then, we did a post-therapeutic dosimetry using the same software (Simplicit90Y™) to confirm the concordance of the pre and post-therapeutic doses. Those 23 patients have been followed up biologically and radiologically for at least six months. The biologic evaluation measured the liver function test, including bilirubin, albumin, ALT, AST, prothrombin time, and serum level of alpha-fetoprotein. We also recorded the most recent lab test before the treatment session and several follow-up measurements. On the other hand, a radiological evaluation was performed early, at 8 to 12 weeks, then at 20 to 24 weeks, whether by a computed tomographic scan (CT) or magnetic resonance imaging (MRI), with IV contrast injection and a dedicated multi-phase liver protocol.

### 4.3. Data Analysis and Response Categorization

Expert radiologists analyzed and compared images before and after SIRT according to the Modified Response Evaluation Criteria in Solid Tumors (mRECIST) which is implemented in order to evaluate the hypervascularized region of the tumor at the arterial phase whether in CT or MRI. In addition, we collected the demographic characteristics, HCC risk factors, previous treatment, and all neoplasm details regarding the tumor size, number, location, morphological characteristics, and vascular invasion. The overall outcome of the radioembolization was based essentially on the mRECIST criteria. The categorization of the tumor response was as follows: a complete response is defined as a complete regression of enhanced(viable) tumors in all target lesions. Partial response is defined as a partial regression by 30% of enhanced tumor in all target lesions. Progressive disease, for patients with a 20% increase in enhanced tumor and stable disease for those who neither qualify in partial response nor in progressive disease.

## 5. Conclusions

TACE is an effective treatment for unresectable HCC and is the first-line treatment for stage B patients with patent portal vein disease, according to BCLC. However, some studies have highlighted its vascular complications that may subsequently reduce the effectiveness of SIRT. The recently published promising results of SIRT make it an excellent alternative treatment. Our study found a potential outcome of SIRT after TACE with an overall objective response of 73.9%. Therefore, it is important to consider SIRT as a valuable modality that produces positive responses even after chemoembolization.

## Figures and Tables

**Table 1:** Patient and tumor characteristics; represented in *n* (%) and median (range).

Age	69	(58–78)
Female	4	17%
Male	19	83%
Liver function status		
Child–Pugh A	21	9%
Child–Pugh B	2	9%
Liver cirrhosis	17	73.9
Alcohol abuse	14	58%
Hepatitis B virus	4	17%
NASH	2	8%
Tumor number		
Unifocal	15	65%
Multifocal	8	33%
Tumor size	30 mm	(12–70)
Portal veinous invasion		
Tumoral PVT	13	54%
Absence of tumoral PVT	5	21%
AFP ng/ml	84.4	(6–51,470)
BCLC		
Stage A	2	8%
Stage B	4	17%
Stage C	17	74%

**Table 2:** Represents 90Y-based dosimetry (median and IQR).

Microsphere Type/Absorbed Tumor Dose	n/(Median and IQR)
SIRT Microsphere	23
SIR-Sphere	15
TheraSphere	8
*Absorbed Tumor Dose*	
SIR-Sphere	268 Gy (107.1–243.2)
For complete and partial responses *n* = 11	167.4 Gy (127.7–243.2)
For stable or progression responses *n* = 4	79.6 Gy (50.7–183.7)
Thera-Sphere	445 Gy (349.5–563.5)
For complete and partial responses *n* = 6	520 Gy (422.5–587.5)
For stable or progression responses *n* = 2	319 Gy (288.5–349.5)

## Data Availability

The dataset used in this study is not publicly available for confidentiality reasons as it is part of clinical care that respects patient privacy. However, these data are available from the corresponding author upon reasonable request.
